# Supplementary material for: FUT8-mediated core fucosylation of receptor APN drives entry of multiple alphacoronaviruses
Source: PLoS Pathog. 2026 May 18;22(5):e1014227. doi: 10.1371/journal.ppat.1014227 (PMC13221147; doi:10.1371/journal.ppat.1014227)
Supplement: S1 Fig — (DOCX) [file ppat.1014227.s001.docx]

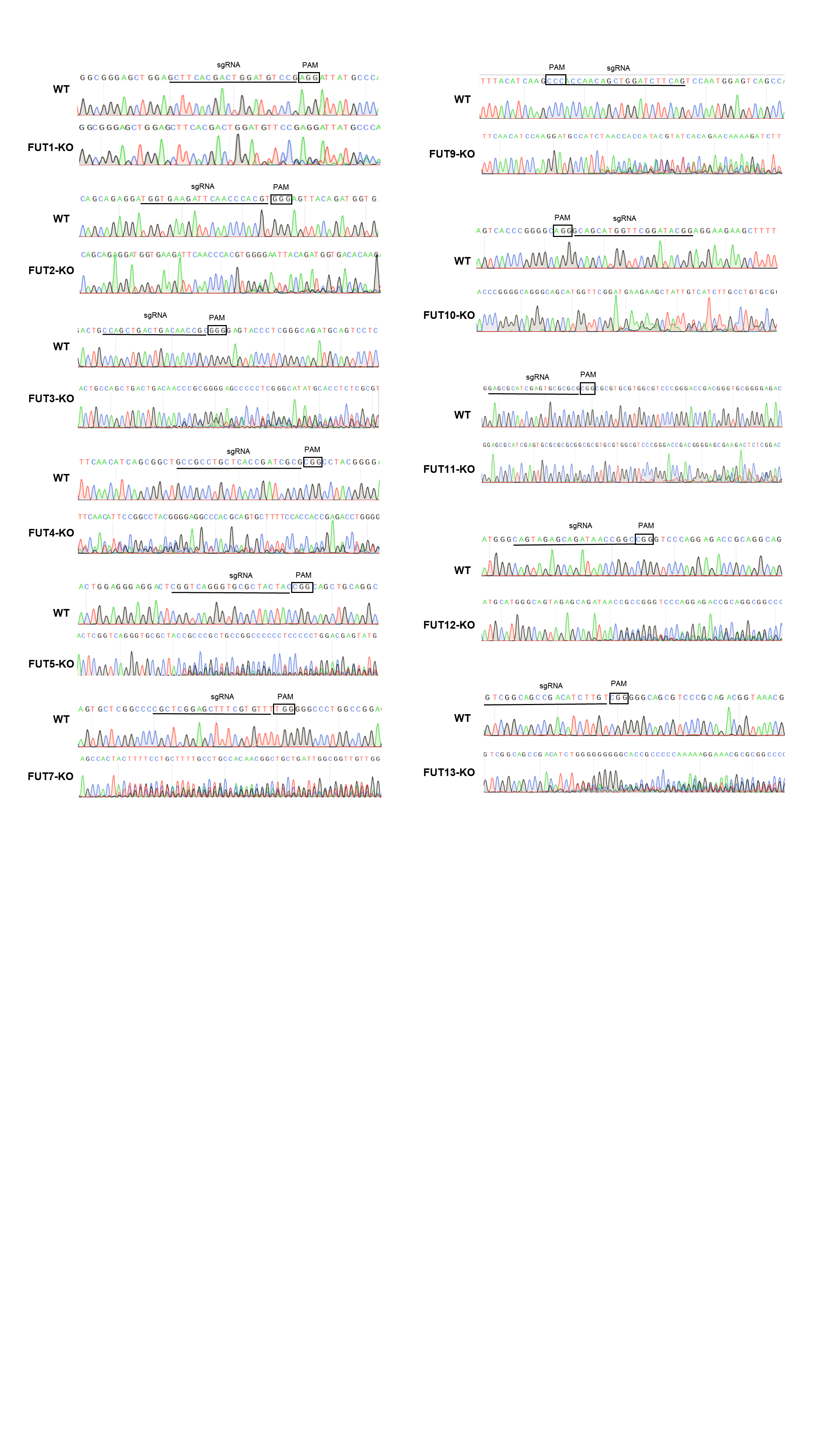


**S1 Fig. Alignment of the nucleic acid sequences of FUT1-13 pooled KO PK-15 cells with those of WT cells respectively. sgRNA-targeted sites are marked by underlines. The PAM sequences are indicated by boxes.**
