# Supplementary figures and images for: FUT8-mediated core fucosylation of receptor APN drives entry of multiple alphacoronaviruses
Source: PLoS Pathog. 2026 May 18;22(5):e1014227. doi: 10.1371/journal.ppat.1014227 (PMC13221147; doi:10.1371/journal.ppat.1014227)

**
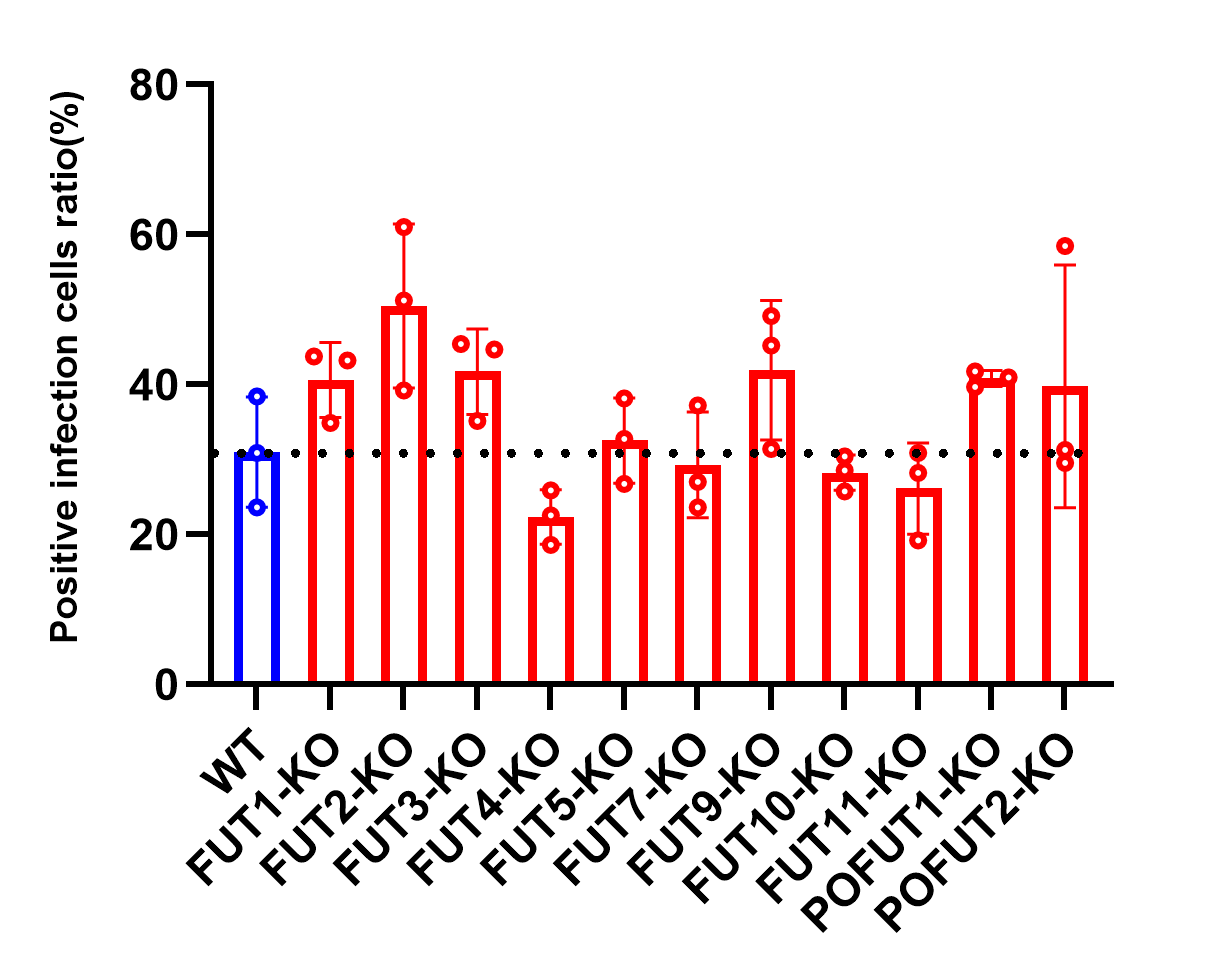
**

**S2 Fig. Quantification of the percentage of TGEV-positive cells shown in Fig. 1H.**

Supplement: S2 Fig — (DOCX) [file ppat.1014227.s002.docx]
