# Supplementary material for: FUT8-mediated core fucosylation of receptor APN drives entry of multiple alphacoronaviruses
Source: PLoS Pathog. 2026 May 18;22(5):e1014227. doi: 10.1371/journal.ppat.1014227 (PMC13221147; doi:10.1371/journal.ppat.1014227)
Supplement: S3 Fig — (DOCX) [file ppat.1014227.s003.docx]

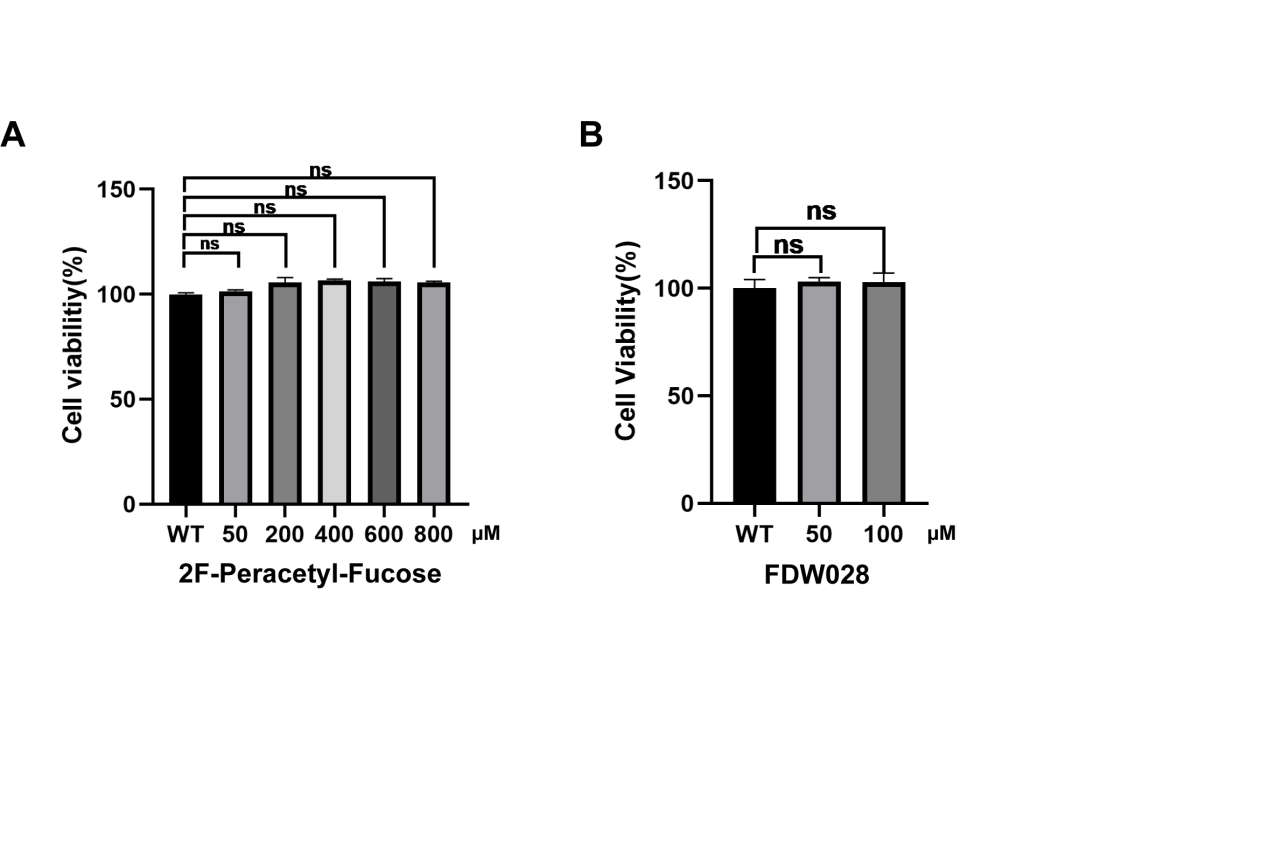


**S3 Fig. PK-15 cells were treated with different concentrations (50, 200, 400, and 800 µM) of 2F-Peracetyl-Fucose for 5 days (A), PK-15 cells were treated with different concentrations (50 and 100 µM) of FDW028 for 2 days (B), and cell viability was assessed by the MTS assay.**
