# Supplementary material for: FUT8-mediated core fucosylation of receptor APN drives entry of multiple alphacoronaviruses
Source: PLoS Pathog. 2026 May 18;22(5):e1014227. doi: 10.1371/journal.ppat.1014227 (PMC13221147; doi:10.1371/journal.ppat.1014227)
Supplement: S4 Fig — (DOCX) [file ppat.1014227.s004.docx]

**
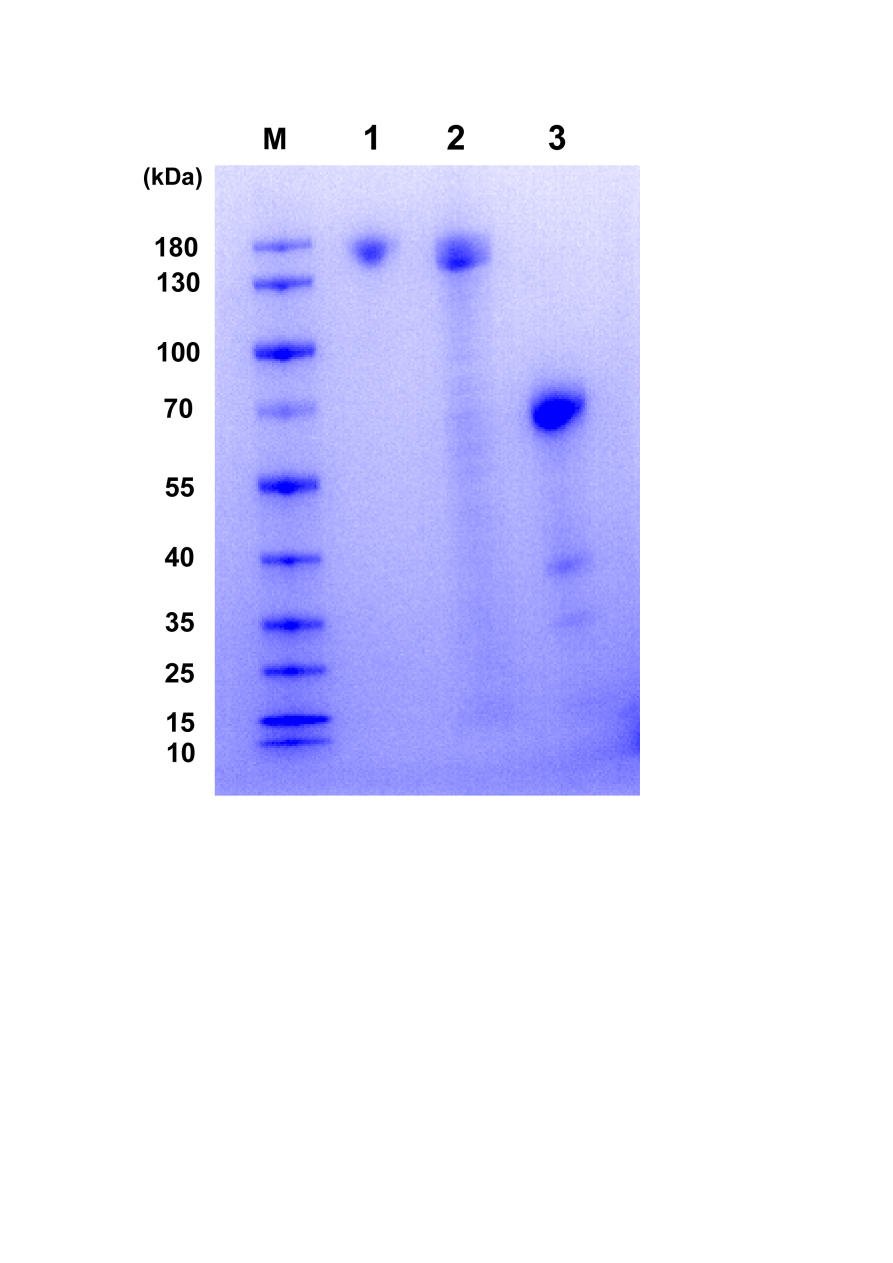
**

**S4 Fig. SDS–PAGE analysis of purified proteins. M: molecular weight marker; Lane 1: Strep-tagged pAPN purifed in FUT8-knockout 293F cells; Lane 2: pAPN purifed in wild-type 293F cells; Lane 3: Fc-tagged TGEV RBD.**
