# Supplementary material for: FUT8-mediated core fucosylation of receptor APN drives entry of multiple alphacoronaviruses
Source: PLoS Pathog. 2026 May 18;22(5):e1014227. doi: 10.1371/journal.ppat.1014227 (PMC13221147; doi:10.1371/journal.ppat.1014227)
Supplement: S5 Fig — (DOCX) [file ppat.1014227.s005.docx]

**
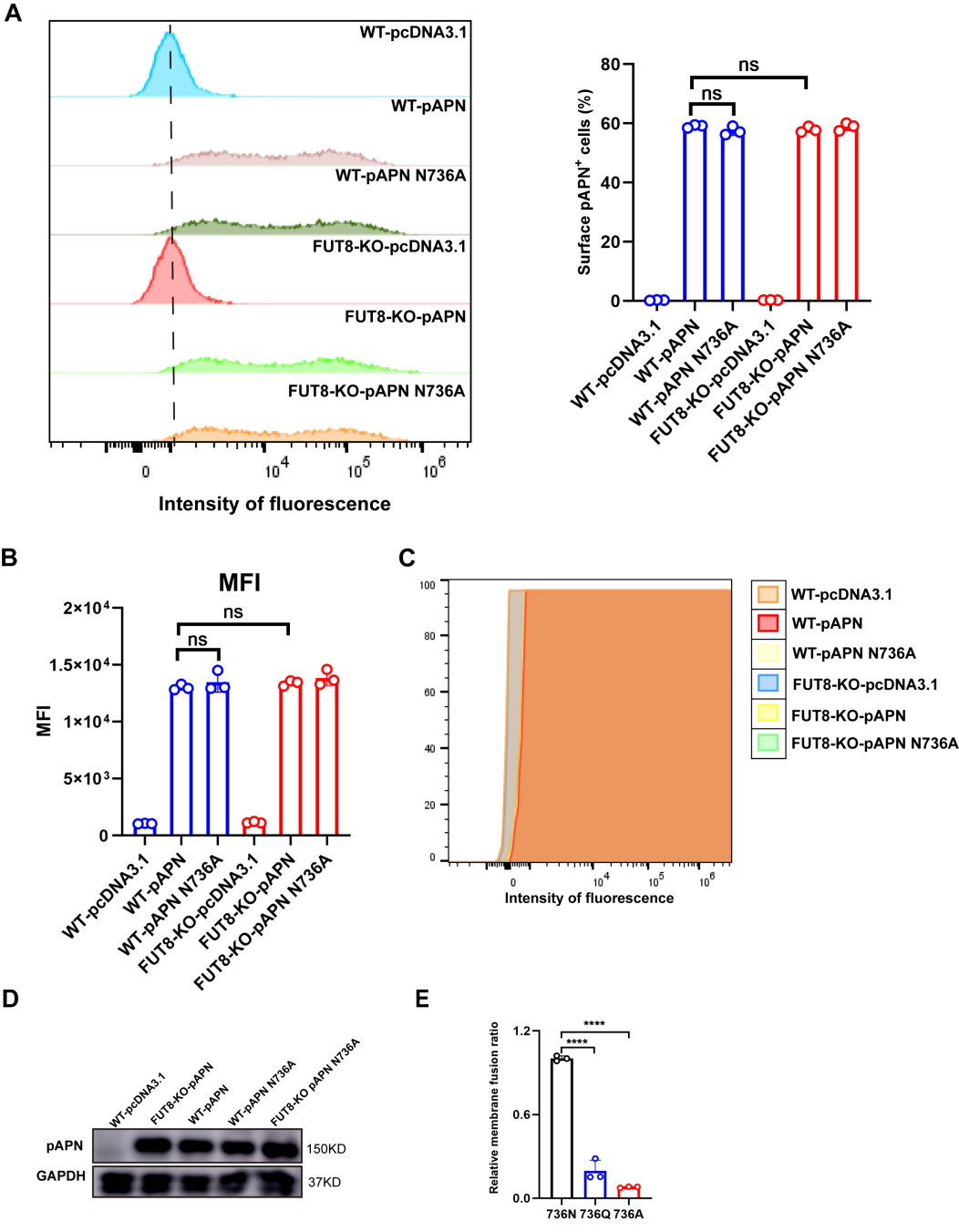
**

**S5 Fig. (A-C) Flow cytometry analysis of cell surface expression of pAPN and N736A pAPN in 293T-WT and 293T-FUT8-KO cells. (D) Western blot analysis of pAPN and pAPN N736A expression in 293T-WT and 293T-FUT8-KO cells. (E) Membrane fusion assay of TGEV spike-mediated fusion in WT pAPN and N736Q/N736A mutants.**
