# Supplementary material for: FUT8-mediated core fucosylation of receptor APN drives entry of multiple alphacoronaviruses
Source: PLoS Pathog. 2026 May 18;22(5):e1014227. doi: 10.1371/journal.ppat.1014227 (PMC13221147; doi:10.1371/journal.ppat.1014227)
Supplement: S6 Fig — (DOCX) [file ppat.1014227.s006.docx]

**
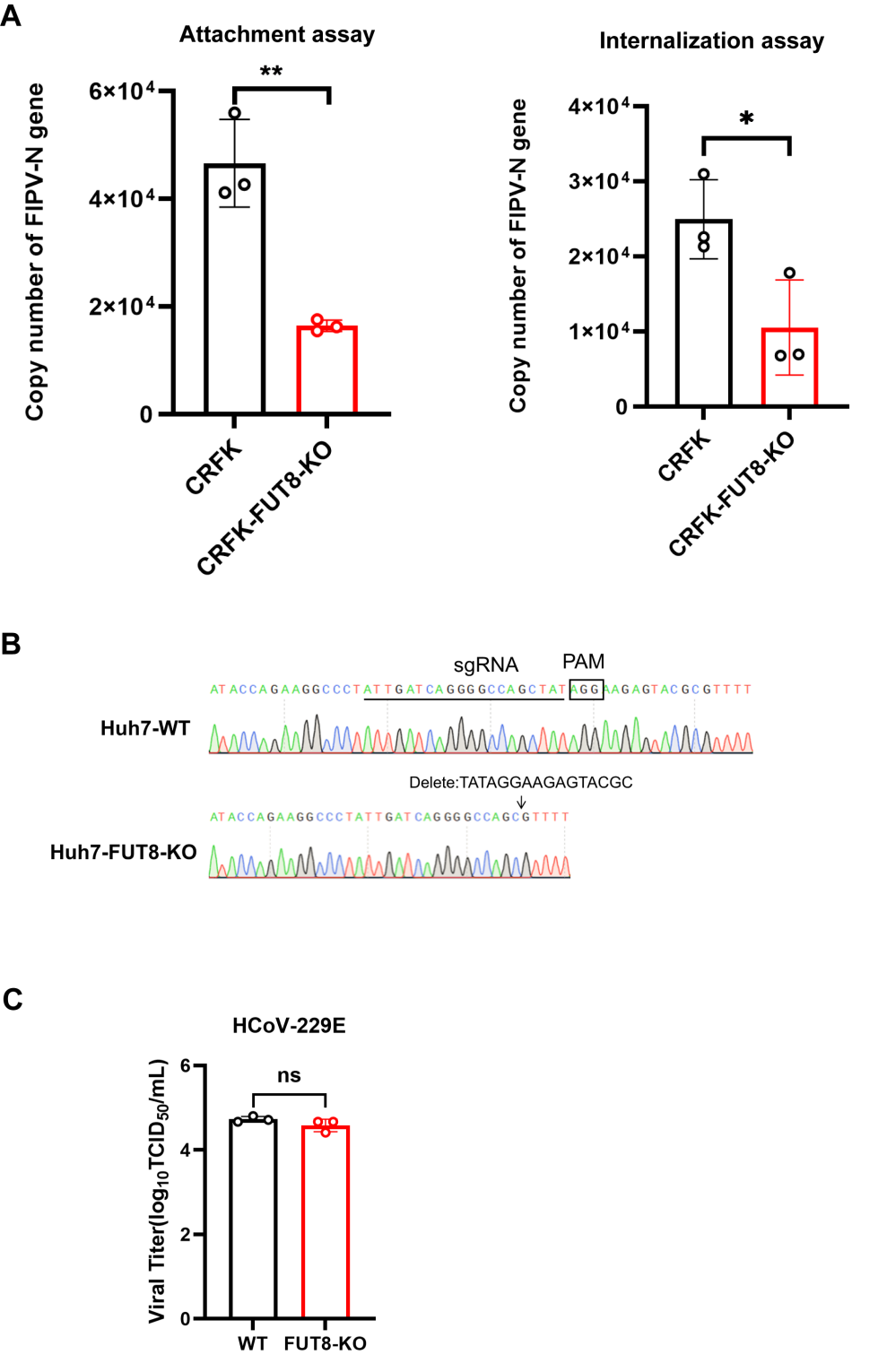
**

**S6 Fig. (A) Viral adsorption and internalization assays of FIPV in CRFK-WT and CRFK-FUT8 KO cells. (B) Alignment of the nucleic acid sequences of Huh7-WT and Huh7-FUT8-KO cells. (C) HCoV-229E infection efficiency in Huh7-WT and Huh7-FUT8-KO cells.**
