# Supplementary material for: FUT8-mediated core fucosylation of receptor APN drives entry of multiple alphacoronaviruses
Source: PLoS Pathog. 2026 May 18;22(5):e1014227. doi: 10.1371/journal.ppat.1014227 (PMC13221147; doi:10.1371/journal.ppat.1014227)
Supplement: Attachment — Submitted filename: Plos Pathogens Response.docx [file ppat.1014227.s011.docx]

RESPONSES TO REFEREE’S COMMENTS:

Additional Editor Comments:

COMMENT: This study presents interesting evidence linking core fucosylation (detected by MS) with functional readouts of viral entry/fusion, and identifies the pAPN N736A mutant as reducing both core fucosylation and entry. However, as currently presented, the data do not establish causality between core fucosylation and Spike-pAPN interaction/viral entry, nor do they fully exclude alternative explanations. Specifically, the reduction in entry/fusion observed with N736A may result from structural effects unrelated to glycosylation. In addition, FUT8‑dependent phenotypes could arise through broader modulation of host pathways, as reported (PMID: 38253527).

RESPONSE:

We thank the Editor for pointing out this issue. In the following sections, you will find our responses to each of your points and suggestions.

COMMENT: To address this point, experiments directly measuring Spike/RBD binding to purified pAPN from FUT8‑KO cells (core fucose–deficient) and WT cells (core fucose–competent) are strongly recommended (also raised by Reviewer 3; see below). In addition, testing an alternative substitution, N→Q (glycan removal with minimal backbone perturbation) versus N→A at the implicated site, is recommended to determine whether entry defects track specifically with loss of glycosylation/core fucose rather than residue substitution.

RESPONSE:

We sincerely thank you for your kind suggestion. We directly measuring TGEV-RBD binding to purified pAPN from FUT8‑KO 293F cells and WT 239F cells in Fig. 4A (revised version). The surface plasmon resonance (SPR) assay results showed that WT-pAPN showed strong binding to TGEV RBD with a dissociation constant (KD) of 5.09 nM, whereas FUT8-KO pAPN showed no detectable binding even at higher analyte concentrations. These results clearly illustrate the critical relationship between FUT8-mediated core fucosylation and the interaction between pAPN and TGEV RBD. Although SPR analysis showed that FUT8-KO pAPN failed to bind the TGEV RBD, cell-based assays such as membrane fusion demonstrated that TGEV-S retained partial entry capacity. This discrepancy suggests that additional host factors may contribute to viral entry at the cellular level.

Fig. 4A (revised version)

In addition, following the Editor's suggestion, we introduced an N→Q substitution at residue 736 of pAPN. Notably, the N736Q mutant also showed a significant reduction in viral entry, indicating that glycosylation at this site—rather than structural perturbation caused by residue substitution—is important for TGEV RBD-mediated entry S5E Fig (revised version).

S5E Fig (revised version)

COMMENT: The editorial board also noted technical weaknesses that should be carefully addressed. 1) Results from the cell‑based Spike‑binding assay may be confounded by the gating strategy and pAPN surface abundance. The baselines across panels in Supplementary Fig. S3 appear shifted. Please provide details of the gating strategy and clarify whether these shifts are due to technical issues; correct if necessary. 2) The fluorescence intensity for N736A in both WT and FUT8‑KO appears reduced compared with WT pAPN. Please provide more precise comparisons—mean fluorescence intensity (MFI) and cumulative distribution plots—to assess cell‑surface pAPN abundance in addition to pAPN‑positive cell counts. 3) Please address concerns about the use of LCA, which is not optimal for selective detection of core fucosylation. AOL appears more specific for detecting core fucose than AAL or LCA (PMID: 17383961).

RESPONSE:

We thank the Editorial Board for pointing out this important issue and apologize for any confusion caused by our previous presentation. To address this concern, we have repeated the experiments and performed a more rigorous flow cytometry analysis to evaluate cell-surface pAPN expression following transfection. Specifically, the baselines were defined using a consistent gating strategy across all samples S5A Fig (revised version). In addition, to provide a more accurate assessment of pAPN surface abundance, we now include both mean fluorescence intensity (MFI) and cumulative distribution analyses S5B-C Fig(revised version). These results demonstrate that pAPN surface expression is comparable between WT and FUT8-KO cells, and that the N736A mutation does not affect its cell-surface expression.

Considering that AOL exhibits higher affinity and specificity for core fucose than LCA, we further evaluated the effect of AOL Fig. 3J (revised version). Consistently, AOL inhibited pAPN-mediated membrane fusion in a dose-dependent manner. These results further support the critical role of core fucosylation in TGEV binding to pAPN and viral entry.

S5A-C Fig (revised version)

Fig. 3J (revised version)

Reviewer #1 (Remarks to the Author):

Major points

Human coronavirus 229E also utilizes APN as its cellular receptor. As shown in Figure 6A, the glycosylation site implicated in other coronaviruses is not conserved in 229E. However, the consequence of FUT8 knockout on 229E infection has not been assessed. The authors should either experimentally evaluate the effect of FUT8 deficiency on 229E entry and replication or discuss the potential mechanistic implications in the manuscript.

RESPONSE:

We appreciate the your suggestion. Following your advice, we generated a FUT8-knockout Huh7 cell line S6B Fig (revised version), and found that FUT8 deficiency did not impair HCoV-229E infection S6C Fig (revised version). Notably, the critical region (739–741) in hAPN shown in Fig. 6 lacks a canonical N-linked glycosylation motif (N-X-S/T), suggesting that this site is unlikely to be glycosylated. Previous studies have shown that HCoV-229E engages hAPN through residues located within the N-terminal region of the receptor (aa 260-353), particularly around the β-strand containing residues 287-291[2-4]. Importantly, the introduction of an N-linked glycosylation site at residue 291 abolishes 229E receptor activity, indicating that this region must remain unglycosylated for viral entry. Together, these findings suggest that, unlike α-coronaviruses infecting pigs, cats, and dogs, HCoV-229E engages hAPN through a glycosylation-independent mechanism, therefore does not rely on FUT8. These observations highlight both the conservation and diversity of coronavirus entry strategies that exploit host glycosylation.

S6B-C Fig (revised version)

Minor points

1. For Figures 2C and 2E, the ineffective 50 μM concentration of 2F-Peracetyl-Fucose can be removed. In addition, the manuscript should briefly describe the mechanisms of the two inhibitors.

RESPONSE:

We appreciate your valuable comments, we have removed the ineffective 50 μM concentration of 2F-Peracetyl-Fucose in Fig. 2C and 2E (revised version).We have added a brief description of the mechanisms of the two inhibitors in the revised manuscript

2F-Peracetyl-Fucose functions as a metabolic inhibitor that is converted intracellularly into GDP-2F-fucose and competitively interferes with fucosyltransferases. In contrast, FDW028 is a selective FUT8 inhibitor that directly binds to the FUT8 catalytic pocket and suppresses FUT8-mediated α1,6-core fucosylation.

2. For Figure 3C, viral replication was assessed by BAC transfection in APN-KO and FUT8-APN-KO cells, and no difference was observed. However, the transfection efficiency of the two cell lines is unknown. The author should include a control experiment using a fluorescent or luciferase reporter plasmid to confirm that the transfection efficiencies of two cell lines are comparable.

RESPONSE:

Thank you bringing this issue to our attention. We performed a control experiment by transfecting equal amounts of the RL-TK (Renilla luciferase) reporter plasmid into both cell lines ( Response Fig.1). The resulting RLU values were comparable, indicating that the transfection efficiencies of the two cell lines are similar.

Response Fig.1

3.In Fig. 3G, the Internalization N gene level is unexpectedly lower than Attachment, warranting further clarification.

RESPONSE:

Thank you for pointing out these issues. The attachment assay measures virus particles bound to the cell surface, whereas the internalization assay detects virus particles that have actually entered the cells. In the internalization experiment, acidic PBS-HCl (pH 3.0) at 4 °C was used to remove surface-bound, non-internalized virions. Given that internalization assay was performed at 37 °C for 30 min, it is possible that some virus particles that were bound but not yet fully internalized were removed, which may explain why the N gene level in the internalization assay is lower than that observed in the attachment assay.

Reviewer #2 (Remarks to the Author):

Major points

1. The glycomics analysis in Figure 5 is central to the mechanism. However, the biological significance of the specific glycan structures (e.g., those labeled with GT numbers) is not sufficiently explained. To make this key dataset accessible to a broader audience, please briefly annotate the structural features of the major glycans affected by FUT8 knockout in the figure legend or a supplementary figure. Alternatively, the Results section should summarize how the loss of core fucosylation alters the glycan repertoire at the pAPN N736 site.

RESPONSE:

Thank you bringing this issue to our attention. Due to the complexity of glycan structures, we used GT identifiers to label individual glycans in the figures for clarity and readability. The corresponding detailed glycan compositions and structures are provided in Table 1, where each GT number is annotated with its full glycan description. To improve accessibility, we have clarified this point in the figure legend. And we also revised the results to show that loss of core fucosylation removes fucose while preserving the glycan structure at N736.

2. In Figure 1G, knockout of FUT4 also exhibits a partial inhibitory effect on TGEV infection. This observation somewhat challenges the conclusion regarding the specific and essential role of FUT8. The authors should address this point in the text, offering a plausible explanation for effect of FUT4 (e.g., potential redundancy, off-target effects, or an alternative fucosylation pathway) to clarify the contribution of FUT8.

RESPONSE:

We thank the reviewer for this important point. The human fucosyltransferase (FUT) family can be classified into 4 subfamilies based on linkage type and acceptor specificity, including  (i) the α1,2-FUTs (FUT1, FUT2); (ii) the α1,3/4-FUTs (FUT3–FUT7, FUT9); (iii) the α1,6-FUT (FUT8); and (iv) the POFUTs (POFUT1–POFUT4). Although FUT3–7 and FUT9 all belong to the α1,3/4-fucosyltransferase group, they are not functionally equivalent. Differences in substrate specificity and acceptor recognition result in distinct glycan structures and functions[1]. In this context, FUT4 may exert its effect through specific substrate preference or selective molecular interactions, thereby influencing viral infection. Consistently, FUT8 knockout resulted in a much stronger inhibition of viral replication compared to FUT4 knockout in our experiments. Together, these findings suggest that while other FUT family members, such as FUT4, may have a modulatory role in viral infection, FUT8-mediated core fucosylation plays a predominant role in this process.

To avoid potential misunderstanding, we have revised the manuscript to emphasize the importance of FUT8 while removing statements suggesting its uniqueness.This discussion has been incorporated into the revised manuscript.

3. The role of FUT8 in TGEV infection has been demonstrated primarily in the PK-15 cell line. To solidify the physiological relevance of these findings, validation in additional porcine cell lines or, preferably, primary porcine cells is recommended. Furthermore, extending the analysis to human α-coronaviruses like HCoV-229E (which also uses APN) would significantly broaden the impact of the study and highlight the potential translational relevance of targeting FUT8.

RESPONSE:

We appreciate the your suggestion. Following your advice, we generated a FUT8-knockout ST (Sus scrofa Testis) cell line-one of the susceptible cell lines for TGEV and found that FUT8 deficiency also inhibit TGEV infection obviously Fig. 1G (revised version). Further solidifying the physiological relevance of these findings.

We generated a FUT8-knockout Huh7 cell line and found that FUT8 deficiency did not impair HCoV-229E infection S6B-C Fig (revised version). Notably, the critical region (739–741) in hAPN shown in Fig. 6 lacks a canonical N-linked glycosylation motif (N-X-S/T), suggesting that this site is unlikely to be glycosylated. Previous studies have shown that HCoV-229E engages hAPN through residues located within the N-terminal region of the receptor (aa 260-353), particularly around the β-strand containing residues 287-291[2-4]. Importantly, the introduction of an N-linked glycosylation site at residue 291 abolishes 229E receptor activity, indicating that this region must remain unglycosylated for viral entry. Together, these findings suggest that, unlike α-coronaviruses infecting pigs, cats, and dogs, HCoV-229E engages hAPN through a glycosylation-independent mechanism, therefore does not rely on FUT8. Collectively, These observations highlight both the conservation and diversity of coronavirus entry strategies that exploit host glycosylation.

Fig. 1G (revised version)

S6B-C Fig (revised version)

Minor points

1. Please provide a brief description of the TGEV-BAC system in the Methods section, including the nature of the plasmid (infectious clone or replicon) and a citation if it is from a prior publication.

RESPONSE:

We thank the reviewer for this helpful suggestion. We have added a brief description of the TGEV-BAC system in the Methods section and cited the appropriate reference. The TGEV reverse genetics system used in this study is based on a bacterial artificial chromosome (BAC) containing the full-length cDNA of the TGEV genome (WH-1 strain; GenBank accession number HQ462571). Transfection of this BAC plasmid into susceptible cells enables the recovery of infectious recombinant TGEV. This system has been previously described[5].

2. In Figure 3D, FUT8 knockout inhibits TGEV infection more potently than APN knockout. This intriguing result suggests that FUT8-dependent fucosylation may affect other host factors involved in TGEV adsorption. This point should be acknowledged and discussed. Consequently, the description in line 176 stating the effect of FUT8 KO is "similar to" that in pAPN KO cells should be rephrased to reflect this nuance.

RESPONSE:

We thank the reviewer for this insightful comment. We agree that the stronger inhibition observed in FUT8-KO cells suggests that FUT8-mediated fucosylation may influence additional host factors involved in TGEV adsorption. Accordingly, we have revised the wording in the manuscript to state that “Further investigation showed that FUT8 knockout (KO) markedly inhibited viral adsorption and endocytosis, with the previously generated pAPN-KO cell line serving as the control, and exhibited an even stronger inhibitory effect than pAPN-KO at the viral adsorption stage (Fig. 3D and 3E)”.

3. The term "essential" is used repeatedly to describe the role of FUT8-mediated fucosylation. However, key data (Fig. 3D, 3E) show reduction, not complete ablation, of viral binding/entry upon FUT8 knockout or N736A mutation.

RESPONSE:

We appreciate the reviewer’s valuable suggestion, and to avoid overstatement, we have replaced the term “essential” with “important” throughout the manuscript.

4. For Figure 5B and the description in line 232, please clearly define "intensity of core fucosylation" and specify the quantification method and metrics used in the glycoproteomic analysis.

RESPONSE:

We thank the reviewer for this important comment. In the revised manuscript, we have clarified that the “intensity of core fucosylation” refers to the total MS1 signal intensity of all identified core fucosylated glycopeptides derived from LC-MS/MS analysis. These details have now been added to the legend of Fig. 5B (revised version) to clearly define the quantification metrics used in the glycoproteomic analysis.

5. Line 134 and the Figure 2 legend: "FUT8 promotes coronavirus replication" is not precise. "Replication" typically refers to a specific stage of the viral life cycle (genomic RNA synthesis). As the study focuses on entry, "FUT8 promotes coronavirus infection" is more accurate here.

RESPONSE:

We thank the reviewer for the helpful suggestions. These issues have been addressed in the revised manuscript.

6. The role of FUT8 in other α-coronaviruses (Figure 6) is demonstrated using a cell-cell fusion assay. To provide a more comprehensive validation, direct virus binding and internalization assays could be added.

RESPONSE:

Thank you for raising this question. Because our lab currently has access to infectious FIPV, but not infectious CCoV. Therefore we performed viral adsorption and internalization assays in CRFK-WT and CRFK-FUT8-KO cells. The results demonstrated that FUT8 knockout indeed suppressed both the adsorption and internalization stages of FIPV infection S6A Fig (revised version).

S6A Fig (revised version)

7. Lines 209-212: The statement regarding the conservation of glycosylation sites between cAPN and pAPN is ambiguous. Please rephrase for clarity, explicitly stating that the homologous sites are N736 in pAPN and N747 in cAPN.

RESPONSE:

We thank the reviewer for this helpful suggestion. We have revised the sentence for clarity to explicitly state that the homologous glycosylation sites are N736 in pAPN and N747 in cAPN.

8. Typos and formatting should be corrected throughout the manuscript.

RESPONSE:

Thank you for your suggestions. All of these issues have been addressed in the manuscript.

Reviewer #3 (Remarks to the Author):

Major points

1. No biochemical proof that the N736 glycopeptide from WT cells (not from FUT8-KO cells) is directly recognized by the TGEV spike. An in-vitro binding assay (e.g., SPR, BLI or MST) using synthetic glycopeptides with/without core fucose would fill this mechanistic gap.

RESPONSE:

We sincerely thank the reviewer for this helpful suggestion. To directly evaluate the interaction between TGEV RBD and pAPN, we performed surface plasmon resonance (SPR) assays using purified pAPN derived from WT 293F cells and FUT8-KO 293F cells Fig. 4A (revised version). The results showed that WT pAPN exhibited strong binding to the TGEV RBD with a dissociation constant (KD) of 5.09 nM, whereas pAPN purified from FUT8-KO cells showed no detectable binding even at higher analyte concentrations. These data provide direct biochemical evidence that FUT8-mediated core fucosylation is essential for the interaction between pAPN and the TGEV RBD. Although SPR analysis showed that FUT8-KO pAPN failed to bind the TGEV RBD, cell-based assays such as membrane fusion demonstrated that TGEV-S retained partial entry capacity. This discrepancy suggests that additional host factors may contribute to viral entry at the cellular level.

A study showed that mutation of the threonine residue within the N-linked glycosylation motif (N-X-T) in canine APN (cAPN), specifically T749R, disrupts glycosylation and results in a complete loss of binding to coronavirus RBD in vitro[6]. Importantly, this strategy selectively removes glycosylation without altering the asparagine residue itself, thereby providing a precise approach to dissect glycan-dependent interactions. This result indicates that N-linked glycosylation at cAPN N747 plays a critical role in mediating binding to the viral RBD. Given the conservation of this glycosylation site, it also provides supporting evidence that glycosylation at the corresponding site in pAPN (N736) contributes to receptor-virus interaction.

In addition, we introduced an N→Q substitution at residue 736 of pAPN. Notably, the N736Q mutant also showed a significant reduction in viral entry S5E Fig (revised version). Because this mutation abolishes N-linked glycosylation without substantially altering the peptide backbone, these results further support that glycosylation at residue N736, rather than structural perturbation caused by residue substitution, is critical for TGEV RBD-mediated entry.

Fig. 4A (revised version)

S5E Fig (revised version)

2. The claim that FUT8 is “essential” for α-CoV entry needs qualification with human coronavirus 229E, which also uses APN; its dependence should be examined in human cell lines.

RESPONSE:

Thank you bringing this issue to our attention.We generated a FUT8-knockout Huh7 cell line and found that FUT8 deficiency did not impair HCoV-229E infection Fig. S6B-C (revised version). Notably, the critical region (739-741) in hAPN shown in Fig. 6 lacks a canonical N-linked glycosylation motif (N-X-S/T), suggesting that this site is unlikely to be glycosylated. Previous studies have shown that HCoV-229E engages hAPN through residues located within the N-terminal region of the receptor (aa 260-353), particularly around the β-strand containing residues 287-291[1-3]. Importantly, the introduction of an N-linked glycosylation site at residue 291 abolishes 229E receptor activity, indicating that this region must remain unglycosylated for viral entry. Together, these findings suggest that, unlike α-coronaviruses infecting pigs, cats, and dogs, HCoV-229E engages hAPN through a glycosylation-independent mechanism, therefore does not rely on FUT8.

Collectively, these observations highlight both the conservation and diversity of coronavirus entry strategies that exploit host glycosylation.Accordingly, we have revised the manuscript to indicate that FUT8 is important for the entry of several α-coronaviruses, rather than universally essential for all α-CoVs.

S6B-C Fig (revised version)

Minor points

1. Figure 1G lacks quantification; please provide percentage inhibition for each FUT KO.

RESPONSE:

We thank the reviewer for this valuable suggestion. We have quantified Figure 1G by calculating the percentage inhibition for each FUT knockout, and the data have been included in the S2 Fig (revised version).

2. Typos and grammatical errors (e.g., “mutiple” in Fig 6 legend) need correction throughout.

RESPONSE:

Thank you for your suggestions. All of these issues have been addressed in the manuscript.

3. Fig. 2A shows a schematic of FUT8 enzyme active-site mutations; it should instead illustrate the impact on glycans caused by the absence of FUT8.

RESPONSE:

We appreciate the your kind suggestion. We have revised the schematic model to clarify that FUT8 knockout or mutation of the catalytic site leads to the loss of α1,6-linked core fucosylation on N-glycans.

Reference

1.	Ghirardello, M., et al., *Structure, function, and implications of fucosyltransferases in health and disease.* Nat Commun, 2025. **16**(1): p. 11279.

2.	Li, Z., et al., *The human coronavirus HCoV-229E S-protein structure and receptor binding.* Elife, 2019. **8**.

3.	Wong, A.H.M., et al., *Receptor-binding loops in alphacoronavirus adaptation and evolution.* Nat Commun, 2017. **8**(1): p. 1735.

4.	Li, Y., et al., *Structural Requirements and Plasticity of Receptor-Binding Domain in Human Coronavirus Spike.* Front Mol Biosci, 2022. **9**: p. 930931.

5.	Wang, G., et al., *The N-Terminal Domain of Spike Protein Is Not the Enteric Tropism Determinant for Transmissible Gastroenteritis Virus in Piglets.* Viruses, 2019. **11**(4).

6.	Tortorici, M.A., et al., *Structure, receptor recognition, and antigenicity of the human coronavirus CCoV-HuPn-2018 spike glycoprotein.* Cell, 2022. **185**(13): p. 2279–2291 e17.
