# Supplementary material for: FUT8-mediated core fucosylation of receptor APN drives entry of multiple alphacoronaviruses
Source: PLoS Pathog. 2026 May 18;22(5):e1014227. doi: 10.1371/journal.ppat.1014227 (PMC13221147; doi:10.1371/journal.ppat.1014227)
Supplement: Attachment — Submitted filename: Plos_Pathogens__Response_auresp_2.docx [file ppat.1014227.s012.docx]

RESPONSES TO REFEREE’S COMMENTS:

Additional Editor Comments:

COMMENT: The revised version has significantly improved the clarity and rigor of the manuscript. However, the Editorial Board has identified the following issues that still need to be addressed:

1. Please include a brief description of coronavirus classification and cellular receptors of coronaviruses in the Introduction.

RESPONSE: We thank the reviewer for this helpful suggestion. We have revised the Introduction to include a brief description of coronavirus classification and their cellular receptors.(revised version).

2. As this study focuses specifically on alphacoronaviruses, please revise the title, abstract, and relevant text throughout the manuscript accordingly. The current wording implies that the findings are broadly applicable to all coronaviruses, which overstates the scope of the study. Please observe ICTV rules when using virus taxa names.

RESPONSE:

We thank the Editorial Board for highlighting this issue and apologize for any confusion caused.We have revised the title, abstract, and relevant text throughout the manuscript to explicitly refer to “alphacoronaviruses” and avoid unsupported generalization. We have also updated virus names and taxonomy to comply with ICTV nomenclature guidelines.
