# Supplementary material for: FUT8-mediated core fucosylation of receptor APN drives entry of multiple alphacoronaviruses
Source: PLoS Pathog. 2026 May 18;22(5):e1014227. doi: 10.1371/journal.ppat.1014227 (PMC13221147; doi:10.1371/journal.ppat.1014227)
Supplement: Attachment — Submitted filename: Plos_Pathogens__Response_auresp_3.docx [file ppat.1014227.s013.docx]

Editor Comments:

The authors have addressed only partially the taxonomy nomenclature matter. They reproduce an outdated subfamily of coronaviruses and don't follow ICTV rules on taxa nomenclature (specifically, the use of italic). Please consult the following website https://ictv.global/faqs/names for general guidance on the ICTV rules and https://www.nature.com/articles/s41564-020-0695-z for application of the rules to the family Coronaviridae.

RESPONSE:

We thank the editor for this helpful comment and apologize for the inaccuracy in the previous description of coronavirus taxonomy and nomenclature.

The manuscript has been revised to ensure compliance with ICTV nomenclature guidelines. Specifically, all taxonomic names at the family and genus levels (e.g., Coronaviridae, Alphacoronavirus, Betacoronavirus) are now italicized, and outdated subfamily terminology has been removed to align with the current classification (revised version).
